# Bio-Inspired Photocatalytic Degradation of Humic Acids over TiO_2_- and Ag-Doped TiO_2_-Functionalized Clinoptilolite: Mechanistic Insights into Nature-Mimicking Oxidation Pathways

**DOI:** 10.3390/biomimetics11060388

**Published:** 2026-06-02

**Authors:** Liliana Bobirică, Cristina Modrogan, Constantin Bobirică, Oanamari Daniela Orbuleţ

**Affiliations:** Department of Analytical Chemistry and Environmental Engineering, National University of Science and Technology POLITEHNICA Bucharest, 1-7 Polizu, 060042 Bucharest, Romania; liliana.bobirica@upb.ro (L.B.); cristina.modrogan@upb.ro (C.M.); constantin.bobirica@upb.ro (C.B.)

**Keywords:** photocatalysis, humic acids, biomimetic processes, titanium dioxide, clinoptilolite, Ag-doping

## Abstract

This study investigates the bio-inspired photocatalytic degradation of humic acids using TiO_2_-functionalized clinoptilolite (C–TiO_2_) and Ag-doped TiO_2_ (C–TiO_2_/Ag) under UV irradiation. TiO_2_ acts as an artificial analogue of naturally occurring photoactive mineral phases, while clinoptilolite provides a biomimetic scaffold mimicking mineral–organic interfaces. Ag doping enhances charge separation and promotes reactive oxygen species formation, accelerating degradation. The effects of pH and catalyst composition were evaluated over a range of conditions, including the native pH of the humic solution. Degradation was monitored via changes in UV_254_ absorbance, VIS_436_ absorbance, and COD values, revealing a multistage pathway: rapid decolorization of chromophoric groups, slower breakdown of aromatic structures, and final mineralization. Acidic conditions further enhanced performance through increased adsorption and ROS (reactive oxygen species) generation, while measurable activity persisted at near-natural pH values. Kinetic analysis indicated pseudo-first-order behavior, with the highest apparent rate constants obtained for VIS436 removal under C–TiO_2_/Ag at pH 3 (k = 0.0166 min^−1^), followed by COD_1_ (k = 0.0190 min^−1^), confirming faster oxidation of labile fractions and slower mineralization of recalcitrant intermediates. Therefore, the results demonstrate that semiconductor–mineral hybrid systems can serve as biomimetic platforms that reproduce and accelerate natural self-purification processes, providing mechanistic insights into nature-inspired pathways for water treatment.

## 1. Introduction

Humic substances (HSs), major components of natural organic matter (NOM) in surface and groundwater, are recalcitrant, high-molecular-weight compounds that can act as precursors for disinfection byproducts (DBPs) during water treatment, posing significant environmental and health concerns [1,2,3]. Their complex aromatic and aliphatic structures, combined with functional groups such as carboxyls and phenolics, render them resistant to conventional treatment processes, including coagulation, adsorption, and biological degradation [4,5,6]. Advanced oxidation processes (AOPs), particularly heterogeneous photocatalysis using semiconductor materials, have attracted considerable attention as effective methods for HSs degradation due to their ability to generate highly reactive species such as hydroxyl radicals (•OH), which can non-selectively oxidize organic pollutants [7,8,9].

Titanium dioxide (TiO_2_) is among the most widely investigated photocatalysts, owing to its chemical stability, low cost, non-toxicity, and strong oxidative potential under UV irradiation [10,11,12]. In this context, TiO_2_ can be considered a biomimetic analogue of naturally occurring photoactive mineral phases that drive sunlight-induced photochemical transformations in aquatic environments. However, its intrinsic wide band gap (≈3.2 eV for anatase) limits activity to the UV region, motivating research into metal ion doping or composite formation to enhance charge separation and light absorption. Silver (Ag) doping has been shown to suppress electron–hole recombination and improve reactive oxygen species (ROS) generation, accelerating degradation pathways in a biomimetic fashion [13,14,15,16,17]. Nature-inspired oxidation processes further proceed through multistep pathways involving reactive intermediates formed at interfacial environments, where electron transfer and controlled radical or high-valent metal–oxo species formation enable selective oxidation of complex organic substrates [18]. Similarly, in natural aquatic environments, photoinduced degradation of dissolved organic matter is governed by ROS generated at mineral–water interfaces, leading to sequential transformation of chromophoric structures into progressively more oxidized intermediates [19]. These biomimetic concepts provide a mechanistic framework for semiconductor–mineral hybrid systems, where interfacial charge transfer and ROS-driven reactions govern multistage photocatalytic degradation pathways.

Previous studies on TiO_2_–zeolite photocatalytic systems have shown that the incorporation of zeolitic supports can significantly enhance photocatalytic efficiency by improving the adsorption of organic pollutants and facilitating interfacial charge transfer. As reported in earlier work, the synergy between TiO_2_ and zeolite materials leads to improved degradation rates compared to bare TiO_2_, mainly due to the concentration of pollutants near active sites and reduced electron–hole recombination. These findings provide a relevant framework for the present study, where clinoptilolite is used as a structured mineral support in combination with TiO_2_ and Ag-modified TiO_2_ systems [20,21]. By combining TiO_2_ and Ag with clinoptilolite, the resulting hybrid system reproduces key features of natural self-purification processes while enabling controlled mechanistic studies.

Solution pH critically affects adsorption, surface interactions, and ROS generation. Experiments were conducted across a broad pH range, including the natural pH of the humic acid solution, to provide enhanced mechanistic understanding of multistage degradation pathways, from chromophore decolorization to mineralization.

In this work, we systematically investigate the photocatalytic degradation of humic acids using TiO_2_-functionalized clinoptilolite (C–TiO_2_) and Ag-doped TiO_2_-functionalized clinoptilolite (C–TiO_2_/Ag) under UV-A and UV-C irradiation within a biomimetic framework inspired by naturally occurring mineral–organic interfaces in aquatic environments. In this hybrid system, clinoptilolite acts as a mineral-like support promoting adsorption and interfacial interactions, while TiO_2_ photocatalysis reproduces sunlight-driven oxidative processes involved in the natural transformation of dissolved organic matter. Within this biomimetic approach, Ag incorporation plays a particularly important role by enhancing charge separation, promoting reactive oxygen species generation, and extending the photoresponse toward the UV-A/near-visible region, thereby more closely reproducing the spectral and oxidative conditions characteristic of natural solar-driven photochemical processes. Degradation was monitored via UV_254_ absorbance, VIS_436_ absorbance, and COD measurements, providing complementary insights into structural transformation, decolorization, and mineralization. This integrated approach clarifies the multistep photocatalytic mechanism and highlights the potential of Ag-modified TiO_2_/clinoptilolite hybrid systems as biomimetic platforms for accelerated water self-purification processes.

## 2. Materials and Methods

### 2.1. Materials

The efficiency of humic acid removal from aqueous solutions was investigated using zeolitic materials functionalized with TiO_2_ as photocatalysts. A TiO_2_-functionalized clinoptilolite containing 1 wt% TiO_2_ (denoted C–TiO_2_) was employed under UV-C irradiation (λ < 315 nm). In addition, photocatalytic degradation under UV-A irradiation (300–400 nm) was evaluated using TiO_2_-functionalized clinoptilolite doped with silver (C–TiO_2_/Ag). Natural zeolite (clinoptilolite) was obtained from a commercial source in the Maramureș region, Romania. Its main physicochemical characteristics are summarized in Table 1.

The clinoptilolite contained a minimum of 80% active phase, with a particle size range of 0.25–1 mm and a cation exchange capacity (CEC) of 2.6 meq g^−1^. Humic acid solutions were prepared by dissolving an appropriate amount of a commercial product, Phylon Humic Soluble fertilizer (BCFertilis, Valencia, Spain), derived from leonardite and containing 65 wt% total humic substances, in distilled water. The composition and properties of the commercial humic product are listed in Table 2.

All other reagents used in this study were of analytical grade and purchased from Merck and Fluka. In all experiments, pure water was used.

### 2.2. Preparation of Photocatalysts

The photocatalytic materials were synthesized via a sol–gel method using titanium (IV) isopropoxide (TTIP) as the TiO_2_ precursor. Prior to functionalization, the natural clinoptilolite was pretreated to obtain its sodium form. The zeolite was first treated with HCl solution (2 M) at room temperature for 2 h under continuous stirring, washed with distilled water until pH 5.5, and subsequently contacted with NaCl solution (3 M) for 2 h. After ion exchange, the Na-form clinoptilolite was washed again to pH 5.5 and dried at 105 °C to constant mass.

#### 2.2.1. Synthesis of TiO_2_ Nanoparticles (TiO_2_)

TiO_2_ nanoparticles were prepared by slowly adding 10 mL of TTIP dropwise to 30 mL of absolute ethanol under stirring for 30 min. Subsequently, a nitric acid catalyst solution (3 mL of 65% HNO_3_ diluted in 150 mL distilled water) was added dropwise over 2 h. The resulting suspension was dried at 105 °C for 24 h to remove the solvent and then calcined at 500 °C for 4 h.

#### 2.2.2. Synthesis of Ag-Doped TiO_2_ Nanoparticles (TiO_2_/Ag)

Ag-doped TiO_2_ nanoparticles were synthesized by adding 5 mL of TTIP dropwise to 20 mL of absolute ethanol under stirring for 30 min, followed by dropwise addition of the nitric acid catalyst solution as described above. Separately, 3 g of AgNO_3_ were dissolved in 20 mL of ethanol and 10 mL of 65% HNO_3_. This solution was added to the TiO_2_ sol (a colloidal suspension of TiO_2_ nanoparticles in a liquid medium), and the resulting suspension was processed under the same drying and calcination conditions as for the undoped TiO_2_.

#### 2.2.3. Functionalization of Clinoptilolite

For photocatalyst preparation, 3 g of Na-form clinoptilolite was dispersed in 20 mL of ethanol under stirring. TiO_2_ or Ag-doped TiO_2_ nanoparticles were then added to the zeolite suspension at a loading of 1 wt% relative to the zeolite mass. The mixture was stirred for 1 h, followed by drying at 60 °C for 2 h to evaporate the solvent. The prepared photocatalysts and their corresponding acronyms are listed in Table 3.

### 2.3. Photocatalytic Experimental Setup

**Figure 1:**
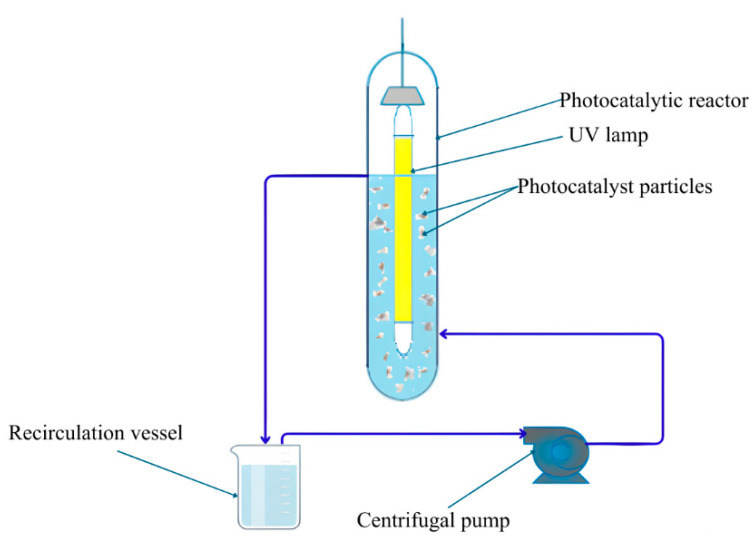
Schematic diagram of the photocatalytic system.

Photocatalytic experiments were performed in a recirculating photocatalytic system, schematically illustrated in Figure 1. The setup consisted of a cylindrical photocatalytic reactor equipped with a centrally positioned UV lamp housed in a stainless-steel sleeve, a centrifugal recirculation pump, and a storage tank for the working solution. UV-C irradiation was provided by a Philips TUV 20 W/T8 lamp (Philips Lighting/Signify, Eindhoven, The Netherlands), while UV-A irradiation was supplied by an OSRAM BLUE UVA 18 W/78 T8 G13 lamp (OSRAM GmbH, Munich, Germany). The incident radiation intensities were measured using a UV meter with a mobile probe (General Tools & Instruments, Secaucus, NJ, USA) and were found to be 2420 μW cm^−2^ for the UV-C lamp and 2430 μW cm^−2^ for the UV-A lamp. For each experimental run, 2 L of humic acid solution (50 mg L^−1^) was prepared, and the photocatalyst was added at a concentration of 1 wt%. The solution was recirculated at a flow rate of 1 L min^−1^ using a centrifugal pump. Samples were collected at predetermined irradiation times, filtered through 0.45 μm Whatman membrane filters (Whatman, Maidstone, UK), and subjected to further analysis.

### 2.4. Analytical Methods

Humic acid degradation was monitored using UV–Vis spectrophotometry and chemical oxygen demand (COD) measurements. UV–Vis spectra were recorded using a Shimadzu UV-1900 spectrophotometer. Maximum absorbance wavelengths were identified at 254 nm (UV_254_) and 436 nm (VIS_436_), corresponding to aromatic/conjugated structures and chromophoric groups, respectively. Quantitative determination of humic compounds was performed using calibration curves obtained in the concentration range of 5–100 mg L^−1^. Degradation efficiency was determined from the relative decrease in the measured analytical signals, namely UV_254_ absorbance, VIS_436_ absorbance, and COD values, with respect to their initial values.

Chemical oxygen demand (COD) was determined according to the APHA standard method 5220 D (closed reflux, colorimetric method) [22]. Briefly, 2 mL of the sample was digested with a potassium dichromate oxidizing mixture in sulfuric acid in the presence of mercury sulfate and silver sulfate as catalysts. Digestion was carried out at 150 °C for 2 h using a thermostated digestion system (Hach Lange LT 200, Hach Lange GmbH, Düsseldorf, Germany). After cooling, absorbance was measured at 620 nm using a Hach Lange DR3800 spectrophotometer. COD values were calculated using a calibration curve prepared with potassium hydrogen phthalate standards in the range of 20–900 mg O_2_ L^−1^. The degradation efficiency (%) was calculated according to the following equation:

$$Degradation\;efficiency\left(\%\right)=\frac{C_{0}-C_{t}}{C_{0}}\times 100$$

where $C_{0}$ represents the initial concentration of humic acids, and $C_{t}$ is the concentration measured at irradiation time t.

The effect of solution pH on photocatalytic degradation was investigated by adjusting the initial pH to 3.0, 7.0, or 9 using HCl or NaOH solutions (0.1 M). pH measurements were performed using an INOLAB pH meter equipped with a SenTix 41 electrode (WTW, Xylem Analytics, Weilheim, Germany). Pure water was used throughout the entire experiment.

### 2.5. Morphological Analysis

**Figure 2:**
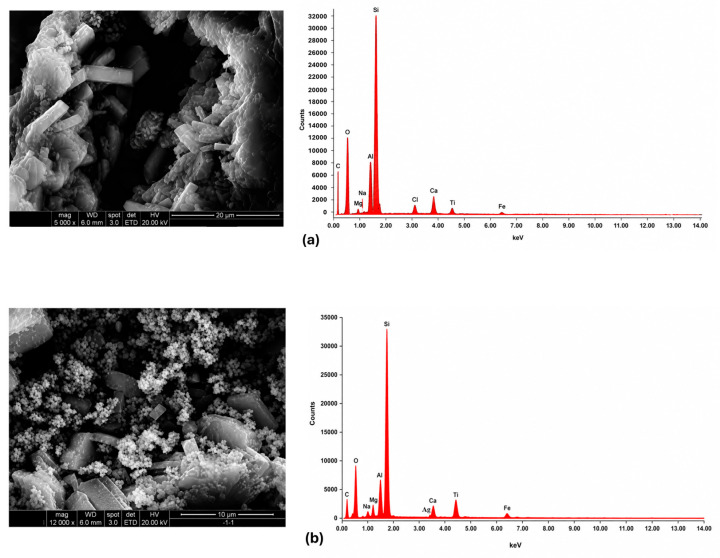
SEM micrographs and EDX elemental analysis: (**a**) C–TiO_2_ photocatalysts; (**b**) C–TiO_2_/Ag photocatalysts.

The morphological features and elemental composition of the prepared clinoptilolite-based photocatalysts (Figure 2) were investigated by SEM and EDX analyses to confirm the successful deposition of TiO_2_ and Ag and to evaluate the structural integrity of the zeolitic support.

SEM analysis of the clinoptilolite–TiO_2_ (Figure 2a) and clinoptilolite–TiO_2_/Ag (Figure 2b) photocatalysts reveals a heterogeneous, porous structure with well-dispersed crystalline aggregates of TiO_2_ (and Ag in the doped system) distributed over the clinoptilolite framework. The zeolitic support maintains its intrinsic porosity, characterized by cavities and channels that favor adsorption and efficient mass transport, while providing a high-surface-area matrix for the dispersion of the active phases. In the Ag-modified sample, a more densely populated and rougher surface is observed, indicating successful incorporation of Ag together with TiO_2_ and enhanced textural development. EDX analysis confirms the presence of the expected elemental composition (Si, Al, O, Ti, and Ag), verifying the successful formation of both composites without significant structural disruption of the zeolite. Ag was introduced during the sol–gel synthesis at a controlled nominal loading, resulting in successful incorporation of silver into the TiO_2_/clinoptilolite composite, as confirmed by EDX analysis. Overall, the combined SEM–EDX results demonstrate the formation of stable, well-integrated photocatalytic materials with preserved porosity and effective distribution of the active phases, suitable for adsorption-assisted photocatalytic applications.

## 3. Results and Discussion

The photocatalytic degradation of humic acids was systematically evaluated using three complementary analytical approaches, namely UV absorbance at 254 nm (UV_254_ absorbance), visible absorbance at 436 nm (VIS_436_ absorbance), and chemical oxygen demand (changes in COD), to discriminate between structural transformation, decolorization, and mineralization pathways. This combined analytical strategy was applied consistently throughout the study, regardless of catalyst composition (C–TiO_2_ or C–TiO_2_/Ag), irradiation wavelength, or solution pH. By integrating TiO_2_ and Ag onto a clinoptilolite scaffold, the system not only accelerates photocatalytic reactions but also mimics natural mineral–organic interfaces and sunlight-driven degradation processes in aquatic environments, providing a biomimetic perspective on humic acid transformation. In all cases, the monitored parameters exhibited a progressive decrease with irradiation time. However, the degradation efficiencies and kinetic trends differed markedly depending on the analytical method employed, clearly highlighting the multistep nature of humic substance photocatalysis.

### 3.1. Photocatalytic Degradation over TiO_2_-Functionalized Clinoptilolite (C–TiO_2_)

**Figure 3:**
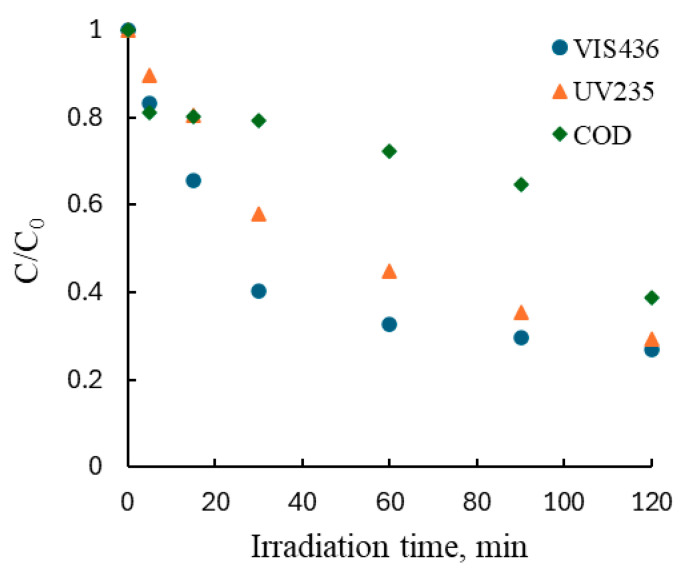
Photodegradation of humic compounds over C-TiO_2_ as a function of irradiation time (UV-C irradiation; pH 8.3).

The photocatalytic performance of C–TiO_2_ toward the degradation of humic compounds was evaluated using an aqueous solution with an initial concentration of approximately 50 mg L^−1^. The measured pH of the solution was 8.3, close to environmentally relevant conditions. Samples were periodically collected under UV-C irradiation and analyzed by UV–Vis spectrophotometry (based on UV_254_ and VIS_436_ absorbance) and chemical oxygen demand (COD) measurements. The experimental results in terms of degradation efficiency are summarized in Table 4 and Figure 3.

The evolution of UV_254_ absorbance, commonly associated with aromatic rings and π-conjugated systems [23], revealed a gradual and continuous decrease in the measured values from 47.8 to 13.96 mg L^−1^, corresponding to ~71% degradation efficiency after 120 min. This progressive decline indicates that photocatalysis primarily disrupts the aromatic backbones and extended conjugated systems of humic macromolecules through hydroxyl radical-mediated oxidation rather than causing abrupt decomposition. The formation of intermediate oxygenated species, such as quinones, phenolic acids, or carboxylates, likely occurs during these initial stages, leading to a steady structural transformation observable through changes in UV_254_ absorbance. By acting on humic substances adsorbed onto the clinoptilolite surface, TiO_2_ facilitates a biomimetic transformation similar to natural photochemical oxidation at mineral–water interfaces.

In contrast, VIS_436_ absorbance (associated with chromophoric groups responsible for solution color) decreased more rapidly, reaching >60% degradation efficiency based on VIS_436_ absorbance within 30 min and ~73% after 120 min. This faster decline reflects the preferential oxidative attack of photogenerated reactive oxygen species (ROS), particularly hydroxyl radicals (•OH) and superoxide anions (O_2_•^−^), on chromophoric moieties such as conjugated carbonyl and aromatic quinone groups [24,25]. The temporal offset between the decreases in VIS_436_ and UV_254_ absorbance suggests that decolorization precedes the complete breakdown of aromatic structures, generating colorless but chemically active intermediates that still contribute to COD values.

The COD profile differed significantly from the optical indicators. During the initial 0–30 min, the reduction in COD values remained below 25%, despite substantial decreases in both UV_254_ absorbance and VIS_436_ absorbance. This lag indicates that early photocatalytic steps primarily involve functionalization and fragmentation of humic macromolecules rather than immediate mineralization. At extended irradiation times, COD reduction increased steadily, reaching ~60% after 120 min, reflecting the gradual mineralization of low-molecular-weight intermediates into CO_2_ and H_2_O.

### 3.2. Effect of Solution pH

**Figure 4:**
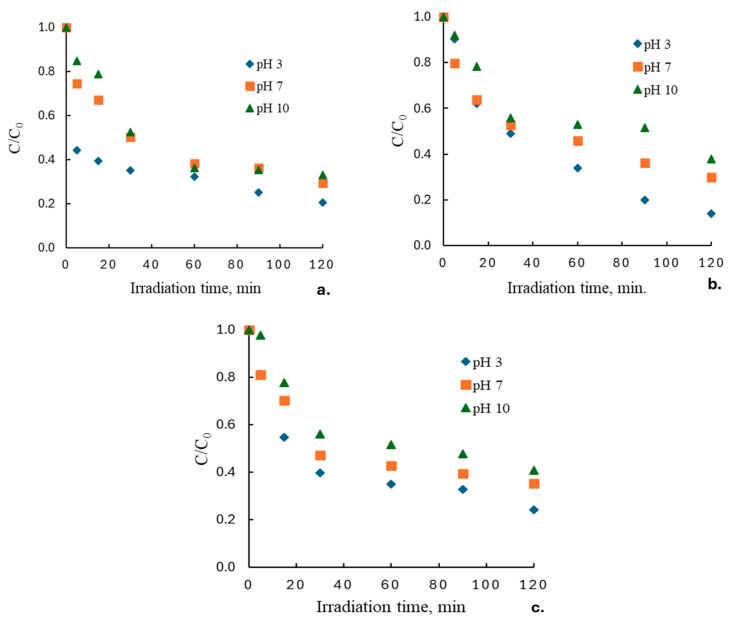
Influence of pH on the photodegradation of humic compounds over C–TiO_2_: (**a**) UV_245_; (**b**) VIS_436_; (**c**) COD.

The influence of solution pH on the photocatalytic degradation of humic compounds was systematically evaluated at pH 3, 7, and 10 using C–TiO_2_ under UV-C irradiation. The degradation efficiencies determined by changes in UV_254_ absorbance, VIS_436_ absorbance, and COD values are summarized in Table 5, while the temporal evolution of humic compound concentration is illustrated in Figure 4. The results clearly demonstrate that acidic conditions significantly enhance photocatalytic performance, whereas neutral and alkaline conditions lead to progressively lower degradation efficiencies.

At pH 3, the photocatalytic process exhibited the highest efficiencies across all analytical indicators. After 120 min of irradiation, degradation efficiencies reached approximately 79% based on UV_254_ absorbance, 86% based on VIS_436_ absorbance, and over 75% based on COD values. The simultaneous decrease in UV254 absorbance, visible color, and COD values indicates that acidic conditions favor not only the breakdown of aromatic and chromophoric structures but also the mineralization of humic substances into low-molecular-weight products and inorganic carbon. The rapid increase in the degradation efficiency evaluated from UV_254_ and VIS_436_ absorbance within the first 30–60 min further suggests enhanced adsorption and fast initial reaction kinetics under acidic conditions. This behavior can be explained by the pH-dependent surface charge of TiO_2_ and the dissociation state of humic substances.

At pH values below the point of zero charge (pH_PZC ≈ 6–6.5 for TiO_2_), the photocatalyst surface becomes positively charged, while humic acids remain predominantly negatively charged due to deprotonated carboxylic and phenolic groups. This electrostatic attraction enhances the adsorption of humic compounds onto the C–TiO_2_ surface, thereby increasing the probability of interfacial electron transfer reactions. Enhanced adsorption is a key factor in heterogeneous photocatalysis, as it promotes efficient interaction between the photogenerated charge carriers and the target pollutants. In addition, acidic conditions promote the generation of reactive oxygen species, particularly hydroxyl radicals (•OH). The higher concentration of H^+^ ions facilitates water oxidation at photogenerated holes (h^+^), leading to increased •OH formation. These highly reactive radicals can non-selectively attack aromatic rings, aliphatic chains, and chromophoric functional groups, accelerating both structural degradation and mineralization processes [26,27]. The strong correlation observed between changes in UV_254_ absorbance, VIS_436_ absorbance, and COD values at pH 3 supports the occurrence of advanced oxidation pathways rather than partial transformation alone.

At neutral pH (pH 7), the degradation efficiencies were moderate, with final degradation efficiencies based on UV_254_ absorbance of approximately 71%, 70% based on VIS_436_ absorbance, and 65% based on COD values after 120 min. Under these conditions, the TiO_2_ surface charge approaches neutrality, reducing electrostatic attraction between the photocatalyst and humic substances. Consequently, adsorption efficiency decreases, and photocatalytic reactions become more dependent on bulk-phase radical reactions [28]. This transition reflects a shift from surface-controlled to diffusion-controlled processes, analogous to natural waters where mineral-assisted transformations are less dominant and photochemical reactions occur predominantly in the dissolved phase. Although effective degradation still occurs, the overall kinetics and mineralization degree are lower compared to acidic conditions.

At alkaline pH (pH 10), the photocatalytic process showed the lowest efficiencies, particularly at short irradiation times. The negatively charged TiO_2_ surface under alkaline conditions results in strong electrostatic repulsion with negatively charged humic compounds, significantly limiting adsorption. Moreover, at higher pH values, hydroxyl radicals are more readily scavenged by excess OH^−^ ions, reducing their availability for oxidative attack on organic matter [29]. This explains the slower degradation rates and the lower reduction in COD values observed throughout the irradiation period, despite some improvement at extended irradiation times.

### 3.3. Photocatalytic Degradation over Ag-Doped TiO_2_ (C–TiO_2_/Ag)

**Figure 5:**
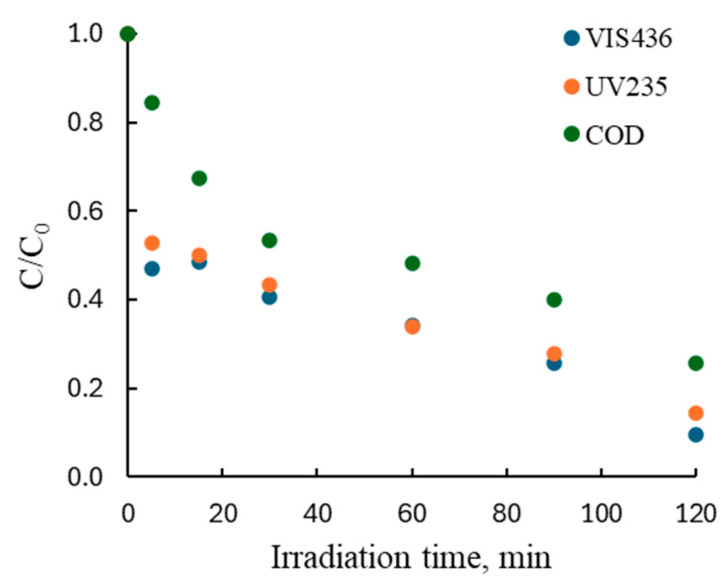
Photodegradation of humic compounds over C–TiO_2_/Ag as a function of irradiation time (UV-A irradiation; pH 3).

Photocatalytic degradation experiments performed with C–TiO_2_/Ag under UV-A irradiation at pH 3 revealed a marked improvement in photocatalytic efficiency compared to the undoped C–TiO_2_ system. The evolution of degradation efficiencies evaluated from UV_254_ absorbance, VIS_436_ absorbance, and COD values as a function of irradiation time is presented in Table 6 and Figure 5. The enhanced performance observed under UV-A irradiation highlights the beneficial role of silver incorporation in extending the photoresponse of TiO_2_ and improving charge carrier utilization. The enhanced photocatalytic activity observed under acidic conditions can be attributed to improved adsorption and increased ROS generation, which is further facilitated by Ag-induced charge separation effects.

After 120 min of irradiation, degradation efficiency based on UV_254_ absorbance reached approximately 86%, while degradation efficiency based on VIS_436_ absorbance exceeded 90%, indicating extensive disruption of aromatic structures and nearly complete elimination of chromophoric groups responsible for visible color. The rapid decrease in VIS_436_ absorbance during the early stages of irradiation suggests that Ag doping significantly accelerates the breakdown of conjugated systems and auxochromic functionalities within humic substances. This behavior consists of an increased generation of reactive oxygen species (ROS) and improved interfacial charge transfer efficiency. Specifically, previous studies have shown that metallic Ag can act as an electron sink due to the formation of Schottky barriers at the Ag/TiO_2_ interface, thereby reducing electron–hole recombination and enhancing reactive oxygen species formation and photocatalytic activity [27,30].

In contrast, the reduction in COD values exhibited a slower but steady increase, reaching approximately 74% after 120 min of irradiation. Although mineralization lagged behind structural degradation and decolorization, the final COD reduction was about 10% higher than that obtained with undoped C–TiO_2_ under comparable experimental conditions. This improvement indicates that silver doping does not merely enhance the cleavage of chromophoric structures but also promotes the oxidation of low-molecular-weight intermediates formed during the photocatalytic process. Such intermediates, often resistant to further oxidation, require sustained ROS availability and efficient charge separation for complete mineralization.

The superior photocatalytic activity of the C–TiO_2_/Ag system can be attributed to several synergistic mechanisms. Silver nanoparticles deposited on the TiO_2_ surface act as effective electron sinks, trapping photogenerated electrons and thereby suppressing electron–hole recombination. This prolongation of charge carrier lifetimes enhances the formation of hydroxyl radicals (•OH) and superoxide radicals (O_2_•^−^), which are the primary oxidizing species responsible for humic substance degradation. Moreover, the localized surface plasmon resonance (LSPR) effect associated with metallic Ag nanoparticles contributes to enhanced light absorption in the UV-A region, enabling efficient photocatalytic activation under longer-wavelength irradiation [31].

Furthermore, the presence of Ag facilitates interfacial electron transfer processes, promoting sequential oxidation pathways. Initially, aromatic and chromophoric moieties are attacked, leading to the formation of hydroxylated and partially oxidized intermediates [32]. These intermediates subsequently undergo ring-opening reactions and further oxidation to smaller aliphatic compounds, which are ultimately mineralized to CO_2_ and H_2_O.

The temporal profiles of changes in UV_254_ absorbance, VIS_436_ absorbance, and COD values clearly demonstrate a hierarchical degradation sequence. Chromophore destruction, reflected by the decrease in VIS_436_ absorbance, occurs most rapidly, followed by structural modification of aromatic moieties indicated by changes in UV_254_ absorbance. Complete mineralization, assessed by COD reduction, remains the rate-limiting step due to the formation and accumulation of recalcitrant intermediate species. This multistage behavior is characteristic of humic acid photocatalysis and underscores the complexity of degrading heterogeneous natural organic matter [33].

### 3.4. Kinetic Analysis and Mechanistic Implications

To describe the kinetics of reactions occurring in heterogeneous solid–liquid systems, the Langmuir–Hinshelwood model is commonly employed. According to this model, the reaction rate (r) depends on the surface coverage (θ) of the catalyst by the organic substrate, linking adsorption phenomena with the subsequent photocatalytic reaction steps [34,35].

(1) r=−dC/dt=kθ

Considering the form of the Langmuir equation:

(2) θ=KC/(1+KC)

and substituting into the above equation yields:

(3) r=−dC/dt=kθ=kKC/(1+KC)

where k represents the reaction rate constant, which is influenced by several parameters such as catalyst loading, photon flux, etc., while K denotes the Langmuir–Hinshelwood adsorption equilibrium constant. Typically, the value of K is obtained from the Langmuir equation based on kinetic studies carried out under irradiation, the results being significantly better than those obtained from dark experiments. C represents the concentration of the organic substrate at time t. Upon integration, the above equation becomes:

(4) $$\ln\left(C_{0}/C\right)+K\left(C_{0}-C\right)=kKt$$

where $C_{0}$ represents the initial concentration of the organic substrate, and t denotes the irradiation time. In the case of dilute solutions, the substrate concentration is low (<10^−3^ mol L^−1^); under these conditions, the term KC is much smaller than 1 and can be neglected, and the reaction follows an apparent first-order kinetics:

(5) $$r=-dC/dt=kKC=k_{obs}C$$

where $k_{obs}$ represents the observed rate constant of the pseudo-first-order reaction. Equation (3) can be simplified to a first-order equation when $C_{0}$ has very low values:

(6) $$-\frac{dC}{dt}=k_{obs}C$$

and it can be simplified into a zeroth-order equation when $C_{0}$ has very large values:

(7) $$\left(C_{0}-C\right)=k_{obs}t$$

The linearized form of the above equation is described by the following equation:

(8) $$\ln\frac{C_{0}}{C}=k_{obs}t$$

By plotting the term $ln(C_{0}/C)$ as a function of irradiation time t, a straight line is obtained whose slope represents the observed rate constant, $k_{\mathrm{obs}}$. Additionally, by plotting the term $\ln\left(C_{0}-C\right)$ against short irradiation times, the region where the reaction follows zero-order kinetics can be identified.

For dilute solutions characterized by low substrate concentrations, photocatalytic degradation generally follows an apparent pseudo-first-order kinetic behavior. Under these conditions, the observed rate constant is commonly used to evaluate the efficiency of the process and can be determined from the temporal variation in substrate concentration. Depending on the initial substrate concentration, the system may exhibit first-order kinetics at very low concentrations or zero-order kinetics at high concentrations, reflecting different surface coverage regimes of the catalyst.

The photocatalytic degradation rate is affected by various factors, such as temperature, solution pH, initial organic substrate concentration, catalyst concentration, light intensity, and the chemical structure of the target compound. However, a major limitation of TiO_2_ is its restricted absorption in the visible region, as it is primarily activated by UV radiation, which limits its efficient use in solar-driven systems.

#### 3.4.1. Photocatalytic Degradation Kinetics of Humic Substances over C–TiO_2_

**Figure 6:**
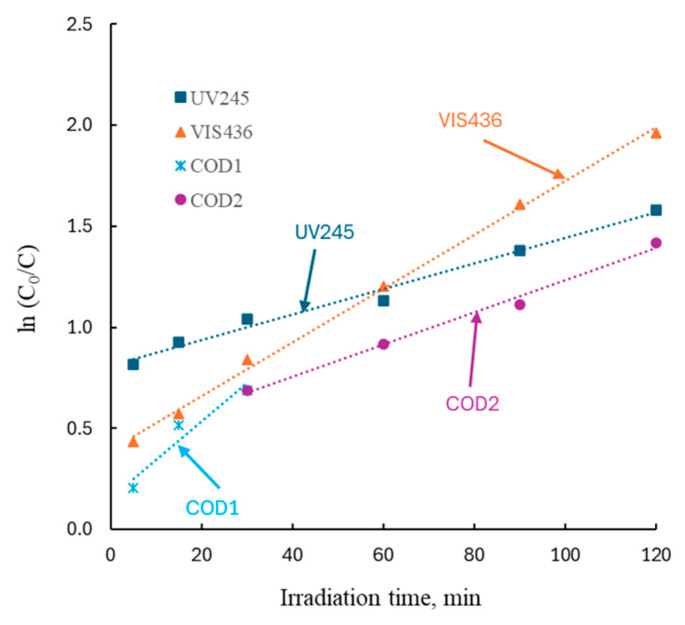
Kinetics of humic acid degradation over C–TiO_2_ photocatalyst (UV-C radiation; pH 3).

The experimental data for the photocatalytic degradation of humic substances over C–TiO_2_ are illustrated in Figure 6. COD_1_ values (the COD component associated with the rapid initial oxidation phase, 0–30 min) exhibit the fastest initial decrease, with a rate constant of 0.0188 min^−1^, slightly exceeding that of VIS_436_ absorbance (0.0133 min^−1^). This suggests that the early oxidation of readily degradable organic components is closely linked to the decolorization of visible chromophores, indicating that a substantial portion of the initial COD reduction corresponds to the breakdown of compounds responsible for visible light absorption.

VIS_436_ absorbance decreases at a rate approximately twice that of UV_254_ absorbance (0.0063 min^−1^), confirming that the degradation of visible chromophores occurs more rapidly than the decomposition of aromatic structures monitored via UV_254_ absorbance at 254 nm. In contrast, COD_2_ values (the COD component corresponding to the slow mineralization phase, 30–120 min) decrease the slowest, with a rate constant of 0.0079 min^−1^, closely matching UV_254_ absorbance decay, reflecting that complete mineralization of the organic matter constitutes the rate-limiting step in the photocatalytic process. Despite the apparent early-time decrease represented by COD_1_, the temporal evolution of COD values (as an overall indicator of organic content) remains slower than the UV_254_ and VIS_436_ absorbance decay observed over the full irradiation period.

These observations are consistent with a sequential, multistage degradation pathway. Initially, the most easily oxidizable fractions react rapidly, contributing both to the reduction in COD_1_ values and chromophore decolorization. This is followed by slower structural transformations, including the degradation of aromatic moieties tracked by UV_254_ absorbance, and ultimately by complete mineralization reflected in COD_2_ values. The close correspondence between COD_1_ values and VIS_436_ absorbance emphasizes that the early stages of the reaction are dominated by the oxidation of highly reactive intermediates, which play a pivotal role in initiating the overall breakdown of humic substances.

The relative differences in reaction rates provide quantitative insight into the temporal evolution of the process. A major portion of COD_1_ reduction can be attributed to chromophore loss, suggesting that the photocatalytic system efficiently targets chromophoric and easily oxidizable fractions at early stages. The slower subsequent stages, as indicated by the lower rates of UV_254_ absorbance decay and COD_2_ decrease, reflect the persistence of more recalcitrant aromatic and aliphatic structures that require prolonged irradiation for full mineralization.

Overall, these results highlight the multistage nature of humic substance photodegradation, where rapid initial reactions are followed by slower structural modifications, culminating in complete mineralization. The strong correspondence between COD_1_ (0.0188 min^−1^) and VIS_436_ absorbance decay (0.0133 min^−1^) provides valuable mechanistic insight into the interplay between total organic matter reduction and chromophore degradation in photocatalytic treatment systems.

#### 3.4.2. Photocatalytic Degradation Kinetics of Humic Substances over C–TiO_2_/Ag

**Figure 7:**
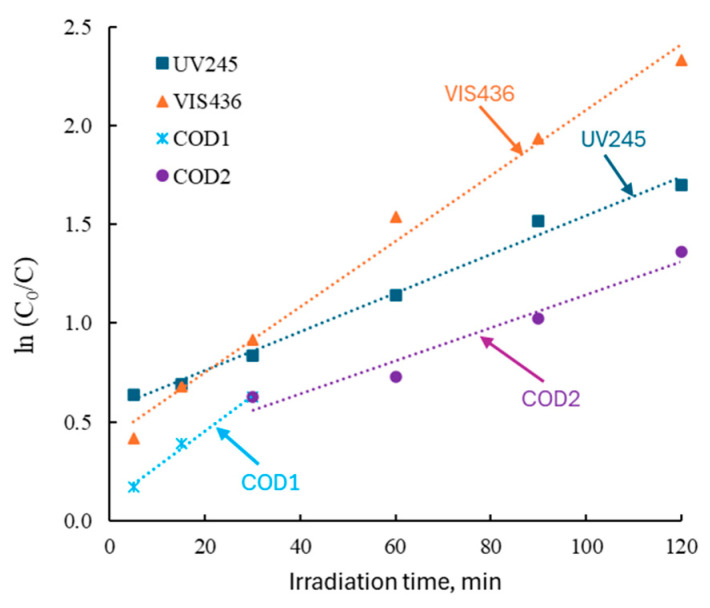
Kinetics of humic acid degradation over C–TiO_2_/Ag photocatalyst (UV-C radiation; pH 3).

In Figure 7, the variation in ln(C_0_/C) with irradiation time is presented for UV_254_ absorbance, VIS_436_ absorbance, COD_1_ values, and COD_2_ values, highlighting the kinetic features of humic substance degradation over the C–TiO_2_ photocatalyst.

COD_1_ values exhibit the fastest initial decrease, with a rate constant of 0.0190 min^−1^, confirming the rapid oxidation of readily degradable organic fractions in the early stages of photocatalysis. A similarly fast response is observed for VIS_436_ absorbance, which decreases with a rate constant of 0.0166 min^−1^. This close correspondence suggests that a significant part of the initial COD reduction is associated with the destruction of visible-light-absorbing chromophores.

In contrast, UV_254_ absorbance decreases more slowly, with a rate constant of 0.0098 min^−1^, indicating that aromatic structures absorbing at 254 nm are more resistant to photocatalytic attack. COD_2_ values show the slowest decrease, with a rate constant of 0.0084 min^−1^, comparable to that of UV_254_ absorbance decay. This behavior suggests that the mineralization of persistent organic matter and the breakdown of stable aromatic moieties occur predominantly in the later stages of irradiation and represent the rate-limiting step of the overall process.

Taken together, these results support a multistage degradation mechanism. The initial stage is dominated by the rapid oxidation of easily reactive compounds and chromophores, reflected by the higher rate constants for COD_1_ values and VIS_436_ absorbance decay. This is followed by slower structural transformations of recalcitrant organic fractions, as indicated by the lower rate constants for UV_254_ absorbance decay and COD_2_ values. The relative differences between the kinetic constants provide quantitative insight into the temporal evolution of humic substance photodegradation on C–TiO_2_, emphasizing the transition from fast initial reactions to slower mineralization-controlled processes.

A comparison between the two photocatalytic systems clearly demonstrates the superior photocatalytic performance of the C–TiO_2_/Ag system, as evidenced by the higher kinetic rate constants. For all monitored analytical indicators (COD_1_, VIS_436_, UV_254_, and COD_2_), the pseudo-first-order rate constants obtained in this study are increased relative to those reported for C–TiO_2_, indicating a general acceleration of the degradation process.

The most pronounced enhancement is observed for the fast-reacting fractions, particularly COD_1_ values and VIS_436_ absorbance decay, whose higher rate constants reflect a more efficient oxidation of readily degradable organic matter and visible chromophores in the presence of Ag. This suggests that silver modification promotes charge separation and increases the availability of reactive species, thereby intensifying the early-stage photocatalytic reactions. Although UV_254_ absorbance decay and COD_2_ values still exhibit lower rate constants compared to COD_1_ and VIS_436_, their values are also higher than those obtained with C–TiO_2_ alone, confirming that Ag incorporation improves not only initial degradation but also the slower transformation of aromatic structures and mineralization steps.

It should be noted that the kinetic representation in Figure 5 and Figure 6 suggests an observed difference in the temporal evolution of the monitored analytical indicators. UV_254_ absorbance and VIS_436_ absorbance exhibit well-defined linear trends over the entire irradiation period, indicating a relatively consistent degradation behavior of aromatic and chromophoric fractions. In contrast, COD values show a more complex temporal profile. The early-time data (0–30 min), referred to as COD_1_, indicate a faster initial decrease; however, this estimation is based on a limited number of experimental points and should therefore be considered as a qualitative indication of early-stage reactivity rather than a strictly resolved kinetic regime. At the later stage (30–120 min), COD_2_ values display a more gradual and linear behavior, reflecting slower transformation processes associated with more resistant intermediates.

To enable a comprehensive comparison of the photocatalytic performance, the apparent pseudo-first-order kinetic rate constants (k) and the corresponding half-life values (t_1/2_ = ln2/k) were calculated for all investigated systems. The results are summarized in Table 7, together with representative literature data for TiO_2_/zeolite- and Ag–TiO_2_-based photocatalysts, allowing for direct evaluation of degradation kinetics across different material architectures and reaction conditions.

C–TiO_2_ and C–TiO_2_/Ag exhibit higher or comparable apparent rate constants than most TiO_2_/zeolite-based systems, despite the higher complexity of humic acid as a target pollutant. The addition of Ag results in consistently improved kinetics and reduced half-life values, confirming enhanced charge separation and increased reactive oxygen species formation. Overall, Ag–TiO_2_ systems reported in the literature show the highest kinetic constants, while zeolite-supported TiO_2_ systems generally display lower activity due to mass transfer limitations and reduced photoresponse.

Overall, the results demonstrate that the photocatalytic performance of TiO_2_-functionalized clinoptilolite (C–TiO_2_) and Ag-doped C–TiO_2_/Ag systems is strongly influenced by interfacial, photochemical, and spectral factors that are also relevant in natural aquatic environments. The observed pH dependence highlights the role of surface charge and adsorption processes, reflecting conditions encountered in mildly acidic and alkaline waters, where mineral–organic interactions and oxidative reactivity differ significantly. In this context, clinoptilolite-based TiO_2_ composites act as simplified analogues of naturally occurring mineral–organic interfaces, where sunlight-driven radical chemistry contributes to the transformation and attenuation of dissolved organic matter.

The incorporation of Ag further enhances photocatalytic activity by improving charge separation, extending the photoresponse toward the UV-A/near-visible region, and increasing the generation of reactive oxygen species, thereby enabling more efficient utilization of lower-energy irradiation comparable to natural solar conditions. This leads to a shift from a partial transformation of chromophoric and aromatic structures toward deeper oxidation pathways, including more effective mineralization of intermediate species.

Kinetic analysis reveals a clear multistage degradation sequence, characterized by rapid initial photobleaching of chromophoric dissolved organic matter (CDOM), followed by slower structural transformation of aromatic and recalcitrant fractions, and ultimately by mineralization. This hierarchical behavior is consistent with established patterns of natural photochemical processes, where fast photobleaching is followed by progressive oxidative aging and long-term degradation of more resistant organic matter. Such stepwise transformation reflects the interplay between reactive intermediates formed during early irradiation stages and subsequent oxidation pathways governing system evolution [30,41].

Therefore, both C–TiO_2_ and C–TiO_2_/Ag systems reproduce key aspects of natural self-purification mechanisms in aquatic environments, where sunlight-driven reactions progressively convert complex organic matter into simpler, more oxidized forms. The enhanced performance of the Ag-modified system further strengthens its biomimetic relevance, demonstrating a more efficient analogue of natural photochemical processes capable of reproducing both rapid phototransformation and slower mineralization pathways.

## 4. Conclusions

The photocatalytic degradation of humic acids over TiO_2_-functionalized clinoptilolite (C–TiO_2_) and Ag-doped TiO_2_/clinoptilolite (C–TiO_2_/Ag) follows a hierarchical, multistage mechanism, starting with rapid decolorization of chromophoric groups, followed by slower breakdown of aromatic structures and eventual mineralization. Experiments conducted across a broad pH range, including the natural pH of the humic acid solution, revealed that acidic conditions (pH 3) enhance degradation through increased adsorption and reactive oxygen species (ROS) generation, while measurable activity persists at near-natural pH values, indicating that the underlying mechanisms are relevant to environmental self-purification processes. Ag doping improves photocatalytic performance by facilitating electron–hole separation, extending light absorption into the UV-A range, and accelerating both early-stage oxidation of labile fractions and later-stage mineralization of recalcitrant intermediates. Kinetic analysis confirmed pseudo-first-order behavior at low substrate concentrations, with faster rates for COD_1_ values and VIS_436_ absorbance decay and slower rates for UV_254_ absorbance decay and COD_2_ values, highlighting the rate-limiting role of mineralization. Overall, the integration of TiO_2_/Ag with clinoptilolite provides a biomimetic platform that reproduces and accelerates natural photochemical self-purification mechanisms, offering mechanistic insights into the sequential transformation of humic substances in aqueous environments.

## Figures and Tables

**Table 1:** Characteristics of the natural zeolite (clinoptilolite) used in the experiments.

Characteristic	Value; % (by Weight)
**Active substance**	min. 80% clinoptilolite
**Moisture**	max. 6%
**Particle size fractions**	0.25–1 mm
**CEC (cation exchange capacity)**	2.6 meq/g
**SiO_2_**	67.42%
**Al_2_O_3_**	11.22%
**Fe_2_O_3_**	0.9%
**CaO**	2.09%
**MgO**	0.72%
**K_2_O**	2.8%
**TiO_2_**	0.17%

**Table 2:** Characteristics of the commercial product “Phylon Humic Soluble Fertilizer” used in the experiments for the preparation of humic compound solutions.

Composition	Value; % (by Weight)
**Appearance**	Granules, dark brown color
**Total humic extract**	min. 65%
**Humic acid**	60%
**Fulvic acid**	5%
**K_2_O (soluble)**	8%
**Solution pH (1%)**	9
**Heavy metal content**	Class A (below the limits set by standards)

**Table 3:** Photocatalytic materials used in the experiments.

Type of Photocatalytic Material	Acronym
**Clinoptilolite functionalized with TiO_2_, 1 wt.%**	C–TiO_2_
**Clinoptilolite functionalized with TiO_2_ and doped with silver ions**	C–TiO_2_/Ag

**Table 4:** Effect of irradiation time on degradation efficiency evaluated by UV_254_, VIS_436_, and COD measurements over C–TiO_2_.

Irradiation Time (min)	Degradation Efficiency (%)		
	UV_254_	VIS_436_	COD
**0**	-	-	**-**
**5**	10.40	16.6	**19.02**
**15**	19.6	34.5	**19.74**
**30**	42.2	59.7	**20.66**
**60**	55.1	67.4	**27.76**
**90**	64.5	70.3	**35.36**
**120**	**70.85**	**73.1**	**61.12**

**Table 5:** Effect of solution pH on degradation efficiency evaluated by UV_254_, VIS_436_, and COD measurements over C–TiO_2_ as a function of irradiation time.

pH	Irradiation Timp (min)	Degradation Efficiency (%)		
		UV_254_	VIS_436_	COD
**3**	0	-	-	-
	5	55.72	9.64	18.58
	15	60.48	37.80	45.33
	30	64.70	50.96	60.21
	60	67.70	66.03	64.83
	90	74.83	80.02	67.07
	120	79.45	85.92	75.78
**7**	0			
	5	25.50	20.10	18.74
	15	32.90	36.12	29.74
	30	49.71	47.39	52.68
	60	61.80	54.21	57.19
	90	63.62	61.62	60.59
	120	70.60	70.13	64.69
**10**	0			
	5	15.12	7.89	2.28
	15	21.20	23.60	22.20
	30	47.46	45.71	43.79
	60	63.65	48.24	48.19
	90	64.47	49.68	52.12
	120	66.77	63.01	59.01

**Table 6:** Effect of irradiation time on degradation efficiency evaluated by UV_254_, VIS_436_, and COD measurements over C–TiO_2_/Ag.

Irradiation Time (min)	Degradation Efficiency (%)		
	UV_254_	VIS_436_	COD
**0**	-	-	**-**
**5**	47.20	53.07	**15.63**
**15**	49.95	51.35	**32.48**
**30**	56.70	59.34	**46.50**
**60**	66.17	65.92	**51.76**
**90**	72.10	74.17	**60.06**
**120**	**85.69**	**90.30**	**74.40**

**Table 7:** Representative literature kinetic values for C–TiO_2_ and C–TiO_2_/Ag photocatalysis (pseudo-first-order model).

Photocatalyst	Pollutant	k (min^−1^)	t_1/2_ (min)	Conditions	Reference
C–TiO_2_	Humic acid	0.0063–0.0188	36.8–110	UV-C	This work
TiO_2_/clinoptilolite	Methylene blue	0.006–0.014	50–116	UV	[36]
TiO_2_/zeolite	Phenol	0.004–0.011	63–173	UV-A	[37]
TiO_2_/zeolite	Organic pollutants (general)	0.003–0.015	46–231	UV	[38]
TiO_2_/zeolite composites	Azo dyes	0.005–0.012	58–138	UV	[39]
C–TiO_2_/Ag	Humic acid	0.0084–0.0190	36.5–82.5	UV-A	This work
AgBr–TiO_2_	Methyl orange	0.012	57.8	UV	[40]
Ag–TiO_2_	Rhodamine B	0.018	38.5	UV-A	[30]
Ag–TiO_2_	Phenol	0.009	77.0	UV	[39]
Ag–TiO_2_	Methylene blue	0.021	33.0	UV	[38]

## Data Availability

The original contributions presented in this study are included in the article. Further inquiries can be directed to the corresponding author.
